# Magnetic Resonance Imaging of Blood Brain/Nerve Barrier Dysfunction and Leukocyte Infiltration: Closely Related or Discordant?

**DOI:** 10.3389/fneur.2012.00178

**Published:** 2012-12-21

**Authors:** Gesa Weise, Guido Stoll

**Affiliations:** ^1^Department of Neurology, University of WuerzburgWuerzburg, Germany; ^2^Fraunhofer Institute for Cell Therapy and ImmunologyLeipzig, Germany; ^3^Translational Center for Regenerative MedicineLeipzig, Germany

**Keywords:** contrast-enhanced MRI, gadolinium-DTPA, gadofluorine, iron oxide nanoparticles, blood brain barrier, neuroinflammation

## Abstract

Unlike other organs the nervous system is secluded from the rest of the organism by the blood brain barrier (BBB) or blood nerve barrier (BNB) preventing passive influx of fluids from the circulation. Similarly, leukocyte entry to the nervous system is tightly controlled. Breakdown of these barriers and cellular inflammation are hallmarks of inflammatory as well as ischemic neurological diseases and thus represent potential therapeutic targets. The spatiotemporal relationship between BBB/BNB disruption and leukocyte infiltration has been a matter of debate. We here review contrast-enhanced magnetic resonance imaging (MRI) as a non-invasive tool to depict barrier dysfunction and its relation to macrophage infiltration in the central and peripheral nervous system under pathological conditions. Novel experimental contrast agents like Gadofluorine M (Gf) allow more sensitive assessment of BBB dysfunction than conventional Gadolinium (Gd)-DTPA enhanced MRI. In addition, Gf facilitates visualization of functional and transient alterations of the BBB remote from lesions. Cellular contrast agents such as superparamagnetic iron oxide particles (SPIO) and perfluorocarbons enable assessment of leukocyte (mainly macrophage) infiltration by MR technology. Combined use of these MR contrast agents disclosed that leukocytes can enter the nervous system independent from a disturbance of the BBB, and vice versa, a dysfunctional BBB/BNB by itself is not sufficient to attract inflammatory cells from the circulation. We will illustrate these basic imaging findings in animal models of multiple sclerosis, cerebral ischemia, and traumatic nerve injury and review corresponding findings in patients.

## Introduction

An important feature of the brain that sets it apart from other organs is the presence of the blood brain barrier (BBB), a selective barrier to the central nervous system (CNS) that impedes the influx of most compounds from blood to brain. The concept of a BBB dates to the late nineteenth century when Ehrlich (1885) observed that water-soluble dyes injected into the circulation leak into all organs of rodents except for the brain. Since then the image of the BBB changed from a static physical wall into a dynamic interface between the blood and the CNS that controls the supply of nutrients while simultaneously shielding it off from potentially harmful substances. The central anatomical substrate of the BBB is the cerebral endothelium which is characterized by the presence of tight cell–cell junctions (Kniesel and Wolburg, 2000), lack of fenestrations (Fenstermacher et al., 1988), and low pinocytotic activity (Sedlakova et al., 1999). These features restrict paracellular diffusion of water-soluble substances from blood to brain (Hawkins and Davis, 2005). In addition, the BBB is composed of large numbers of pericytes that are embedded into the vascular basement membrane and a layer of astrocytic end-feet ensheathing the vessels (Ballabh et al., 2004). They, together with the endothelial cells, form the so-called neurovascular unit which ensures homeostasis of the CNS microenvironment. Similarly, in the peripheral nervous system (PNS) nerve fibers are protected from the circulation by a blood nerve barrier (BNB).

Impairment of the BBB or the BNB is a critical step in the development and progression of neurological conditions such as ischemic stroke (Latour et al., 2004) and multiple sclerosis (MS; Minagar and Alexander, 2003), but also traumatic brain (Schwaninger et al., 1999) or nerve injury (Seitz et al., 1989). Inflammation is another common hallmark of these disorders. It involves a complex cascade of events in which both, the activation of resident glial cells as well as the infiltration of bone-marrow derived leukocytes play an important role and might impact disease progression. While in ischemic stroke and trauma inflammation occurs as a response to brain or nerve tissue damage, in autoimmune disorders such as MS, inflammation initiates the disease. Hence, in both cases, preservation of BBB/BNB integrity and the prevention of inflammatory responses constitute promising therapeutic targets. However, application of barrier stabilizing anti-edematous or anti-inflammatory treatments requires precise knowledge of the timing and location of BBB/BNB disturbances as well as cellular inflammation.

Conventional magnetic resonance imaging (MRI) that is routinely used to assess CNS and PNS pathologies gives only a gross estimate of tissue damage. Moreover, signal changes are non-specific and do not allow discrimination of areas with edema formation, inflammation, or glial scarring. Recently, novel MR contrast agents helped to gain deeper insights into the pluriformity of neuroinflammation. The present review focuses on the visualization of BBB and BNB disturbances by Gadolinium (Gd)-DTPA and Gadofluorine (Gf) enhanced MRI and depiction of cellular inflammation by iron particle and perfluorocarbon (PFC)-based cellular MRI. By combining these imaging techniques it became apparent that the spatiotemporal relationship between breakdown of the BBB/BNB and leukocyte infiltration is more complex than previously anticipated. To illustrate these issues we will use three paradigmatic disorders of the nervous system as models: ischemic stroke, MS with its animal model, experimental autoimmune encephalomyelitis (EAE), and finally traumatic peripheral nerve injury.

## MR Imaging of BBB and BNB Disturbances

### Conventional Gd-DTPA enhanced MRI

Gadolinium-based MR contrast agents are licensed for a broad range of clinical applications. In the nervous system Gd-DTPA is routinely applied to identify areas with BBB/BNB dysfunction. Upon systemic application it extravasates out of the intravascular compartment at sites with a leaky BBB and accumulates locally leading to a substantial T1-shortening effect within that region. Importantly, signal changes rapidly decline due to a reversal of the diffusion gradient upon clearance of Gd-DTPA from the circulation.

#### Multiple sclerosis

In MS, a chronic inflammatory demyelinating disease of the CNS, Gd-DTPA enhanced MRI is the current gold standard for the evaluation of acute disease activity. However, whereas Gd-DTPA enhancement reliably predicts the occurrence of relapses it does not correlate with cumulative impairment and disability (Filippi et al., 2011). Moreover, as triple-dose Gd-DTPA significantly increased the harvest of enhancing lesions (Silver et al., 2001) it became obvious that conventional Gd-DTPA enhanced MRI only captures a small portion of lesions with a dysfunctional BBB.

#### Ischemic stroke

Ischemic stroke regularly leads to disruption of the BBB which already starts a few hours after the onset of ischemia and lasts for several weeks (Strbian et al., 2008; Brouns and De Deyn, 2009). Interestingly, early parenchymal Gd-DTPA enhancement appears to be a predictor of hemorrhagic transformation in experimental stroke (Knight et al., 1998). Nonetheless, changes of the infusion protocol in rodent studies revealed that subtle changes in BBB permeability are missed by standard dose Gd-DTPA enhanced MRI. Among other reasons this might be due to the greatly lowered blood flow in the affected region that leads to suboptimal delivery of the contrast agent (Nagaraja et al., 2007). Thus, especially in the acute ischemic phase BBB disturbances might escape detection by conventional Gd-DTPA enhanced MRI (Merten et al., 1999).

#### Nerve trauma

As in the brain, nerve injury in the PNS is frequently accompanied by breakdown of the BNB. Nerves undergoing Wallerian degeneration (WD) after transection or crush injury characteristically show a prolongation of the T2 relaxation time at the lesion site and distally in conventional MRI (Does and Snyder, 1996). However, despite extravasation of albumin and Evans blue in experimental studies as clear evidence for BNB opening Gd-DTPA enhancement is not consistently detected in injured nerves (Cudlip et al., 2002; Lacour-Petit et al., 2003). Hence, it cannot be considered as a reliable measure for BNB integrity or dysfunction.

### Gadofluorine enhanced MRI: A novel tool for experimental research

Important proof-of-principle studies with Gf M, a novel experimental MR contrast agent developed by the former Schering AG (Berlin, Germany) revealed that BBB/BNB disturbances are more widespread than previously anticipated and can involve brain areas remote from focal lesions. Gf represents a highly fluorinated Gd-compound originally developed for MR lymphography and imaging of atherosclerotic plaques (Meding et al., 2007). We and others directly compared Gd-DTPA and Gf enhancing lesions in EAE, stroke, and nerve injury and found that Gf detects BBB and BNB disturbances with much higher sensitivity than Gd-DTPA. In these experimental settings Gf enhancement exactly matched the leakage of systemically applied Evans blue, a common marker for albumin extravasation across the BBB. In comparison to Gd-DTPA, Gf has unique molecular binding properties that might explain the different elimination kinetics and its higher sensitivity. Upon intravenous injection Gf is largely bound to serum albumin. It passively diffuses into the nervous system at sites of a leaky BBB or BNB, but, unlike Gd-DTPA gets trapped by local interactions. Thus, Gf persists in lesions long after clearance from the circulation which usually occurs within 24 h after application. Although the binding partners at the molecular level in the CNS and PNS have not yet been elucidated, *in vitro* studies suggested that Gf avidly binds to components of the extracellular matrix (Meding et al., 2007).

#### Experimental autoimmune encephalomyelitis, the animal model of MS

**Figure 1:**
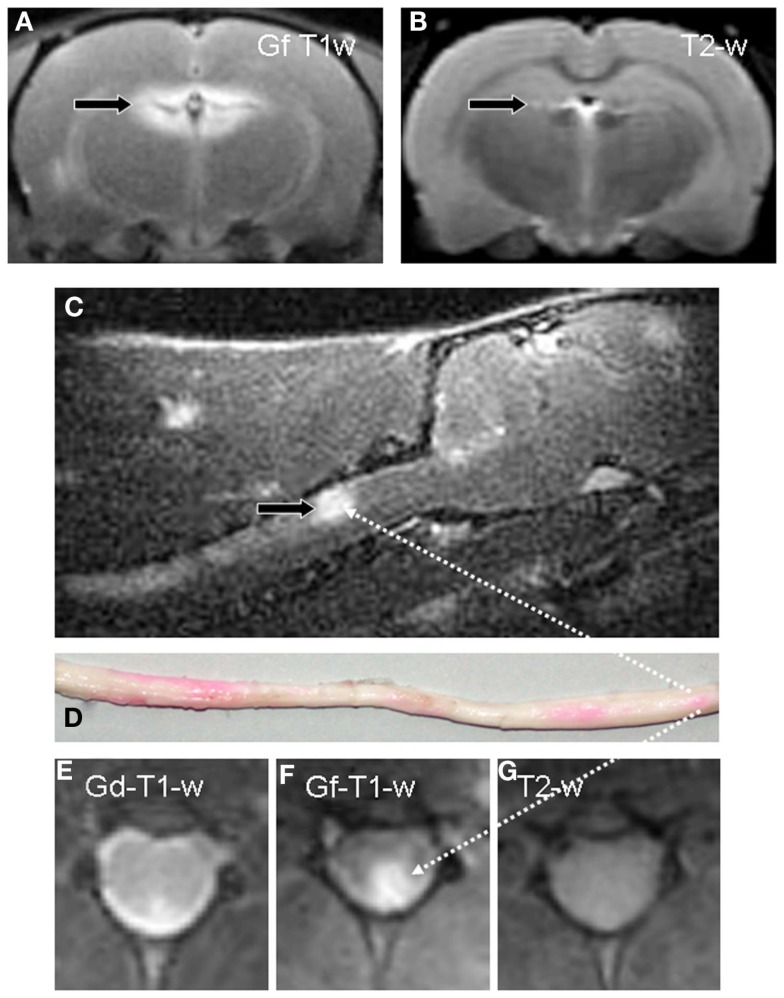
**Gf enhances detection of inflammatory lesions with a dysfunctional BBB in EAE**. A coronal T1-w image of the brain 24 h after systemic application of Gf to a diseased rat displays bright enhancement in the periventricular region **(A)**. By contrast, corresponding coronal T2-w MRI, the standard measure for overall disease burden in MS, shows only a tiny periventricular lesion [arrow in **(B)**]. Sagittal T1-w MRI reveals, amongst other contrast enhancing lesions, a large Gf positive lesion in the dorsal column of the cervical spinal cord [arrow in **(C)**] that is verified on a macroscopic preparation of the entire medulla **(D)**. Foci with Gf deposition appear pink due to the coupling with a carbocyanine dye. Intraindividual comparison of Gf and Gd-DTPA enhancement on coronal slices shows extensive Gf enhancement in the left posterolateral cervical spinal cord **(F)** while Gd-DTPA uptake is far more limited **(E)**. On corresponding T2-w MRI the lesion is indiscernible **(G)**. Adapted from Bendszus et al. (2008).

**Figure 2:**
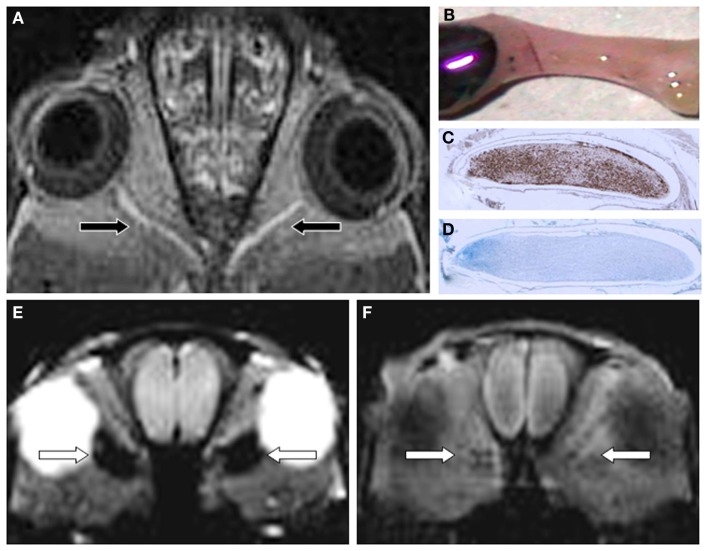
**Gf improves EAE lesion detection in optic nerves (ON) belonging to the CNS (A-D)**. Twenty-four hours after systemic administration of Gf coronal T1-w MRI shows bilateral disturbance of the blood-optic nerve barrier [arrows in **(A)**]. The macroscopic preparation of the left ON **(B)** confirms extravasation of labeled Gf. Thereafter the ON was embedded in paraffin and longitudinal sections were stained for macrophages (ED-1) **(C)** and myelin (Luxol fast blue) **(D)**. Note that the Gf enhancing ON is heavily infiltrated by ED-1 positive macrophages **(C)** and almost completely demyelinated [lack of deep blue staining in **(D)**]. **(E,F)** depict the spatial discrepancy between cellular inflammation and BBB dysfunction in EAE. Coronal CISS (constructive interference steady state) MRI of a severely affected rat 24 h after SPIO application shows strong signal loss in both optic nerves [arrows in **(E)**]. By contrast, the same animal lacks retrobulbar Gf enhancement on corresponding T1-w MRI 24 h after Gf administration **(F)** indicating that macrophages infiltrated the ON without concomitant disturbance of the blood-optic nerve barrier. Since acute macrophage infiltration as shown in **(E)** does not immediately cause leakage of the BBB allowing access of Gf **(F)** we speculate that BBB dysfunction is a secondary and delayed event dependent on previous macrophage infiltration. Adapted from Bendszus et al. (2008) **(A)** and Ladewig et al. (2009) **(E,F)**.

Experimental autoimmune encephalomyelitis is the most widely used animal model for MS. EAE lesions show lymphocyte and macrophage infiltration, variable degrees of demyelination, and leakage of the BBB. EAE lesions can be detected on T2-weighted (w) MRI similar to MS lesions, and partly show Gd-DTPA enhancement on T1-w MRI. When Gd-DTPA- and Gf enhancing lesions were counted, the number of Gf positive EAE lesions by far outnumbered Gd-DTPA positive lesions in individual EAE animals (Bendszus et al., 2008). Moreover, many Gf enhancing lesions were not yet visible on parallel T2-w MRI, the standard sequence to quantify lesion load in MS patients (Figure 1). Thereby, even spinal cord (Figure 1) and optic nerve lesions (Figures 2A–D) that often fail to be visualized despite unambiguous clinical involvement could be depicted by Gf on a standard 1.5 T MR scanner. In a subgroup of animals spinal cord specimens were analyzed for Gf uptake and parenchymal inflammation. Foci of Gf deposition could be detected macroscopically due to the coupling with a carbocyanine dye. Importantly, all tissue specimens devoid of inflammation were Gf negative, while Gf enhancing lesions always exhibited microglial activation or macrophage infiltration. However, a considerable number of lesions with low-grade inflammation failed to show Gf uptake. By contrast, severe inflammation was always accompanied by Gf accumulation. A possible explanation for this observation, besides sensitivity issues, could be that foci with mild microglia activation/macrophage infiltration, but without BBB leakage, represent initial stages of lesion development whereas severe inflammation secondarily evokes BBB opening.

The enhanced sensitivity of Gf for EAE lesion detection was confirmed independently at 7 T high field MRI (Wuerfel et al., 2010). Among a total of 61 contrast enhancing lesions 26 were exclusively visible after Gf administration (nine of them in the optic nerve). The remarkable improvement in lesion detection was accompanied by early Gf uptake in the circumventricular organs (CVO) of diseased animals. The CVO are particular areas of the brain with an incomplete endothelial BBB that participate in immune cell recruitment to the CNS (Schulz and Engelhardt, 2005). Interestingly, Gf mean intensity ratios in the subfornicular organ and the area postrema significantly correlated with disease severity and onset of symptoms suggesting that early Gf enhancement in the CVO might be a predictor of the clinical EAE course.

#### Ischemic stroke

**Figure 3:**
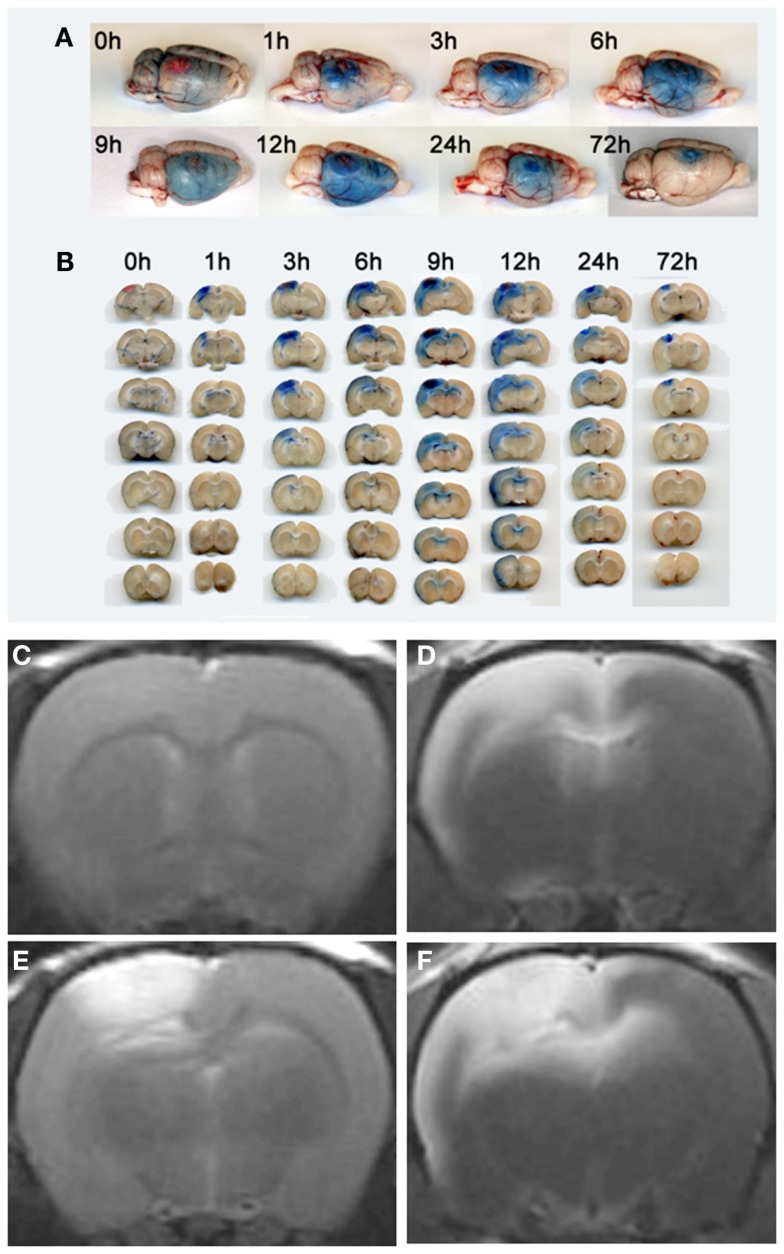
**Visualization of transient BBB opening remote from a photothrombotic lesion by Evans Blue extravasation (A,B), Gd-DTPA (C,E) and Gf enhanced MRI (D,F)**. Rats received Evans Blue i. v. immediately after PT induction or with a delay of 48 h (for 72 h analysis) and were sacrificed at given time points. Total brain preparations **(A)** and coronal brain sections **(B)** of the animals show that Evans Blue extravasation starts at the lesion site, but extends to the remote ipsilateral cortex and the corpus callosum within the first day. Coronal brain slices at the level of the lesion are always shown on top, the other six in the row represent subsequent 1 mm slices located frontally to the lesion **(B)**. At day 3 breakdown of the BBB indicated by Evans blue extravasation is restricted to the PT lesion. **(E)** shows Gd-DTPA enhancement of the photothrombotic lesion within the first 24 h. Note that, in contrast, Gf enhancement occurs within in the lesion, but also affects the ipsilateral cortex and the corpus callosum spared by Gd-DTPA **(F)**. Moreover, T1-w MR images anterior to the PT lesion exhibit no Gd-DTPA **(C)**, but strong Gf enhancement **(D)** according to Evans blue extravasation shown in **(A,B)**. Reproduced from Stoll et al. (2009b).

Similarly, in cerebral ischemia Gf is much more sensitive than Gd-DTPA enhanced MRI. Brain photothrombosis (PT) is a simple model of focal cerebral ischemia that uses local intravascular photo peroxidation to generate highly circumscribed ischemic cortical lesions with almost immediate breakdown of the BBB. Accordingly, PT lesions exhibit an early and strong Gd-DTPA and Gf uptake on T1-w MRI. However, within the first 12 h after the onset of ischemia Gf enhancement, in addition, gradually extended to the entire ipsilateral hemisphere (Stoll et al., 2009b) that undergoes functional alterations, but lacks neuronal damage (Witte and Stoll, 1997). These Gf enhancing areas remote from the lesions did not show Gd-DTPA enhancement. Importantly, the timing of Evans blue extravasation on macroscopic brain slices and histological sections exactly matched the evolution of Gf enhancement on *in vivo* MRI (Stoll et al., 2009b; Figure 3). Several conclusions can be drawn from these observations: (i) Leakage of the BBB is not restricted to structural brain lesions, but also occurs in intact brain areas. Thus, functional states without structural damage can lead to intermittent and fully reversible opening of the BBB. Elucidation of the underlying mechanisms of these transient BBB disturbances might help to develop physiological means to open the BBB for drug delivery. (ii) Lesion-associated BBB disturbances are more abundant in autoimmune disorders of the CNS and ischemic stroke than previously appreciated. Thus, further MR contrast development is warranted since the capabilities of routinely used Gd-DTPA are limited. Fortunately, derivates of Gf are now commercially available for research purposes in animal models.

#### Nerve trauma

**Figure 4:**
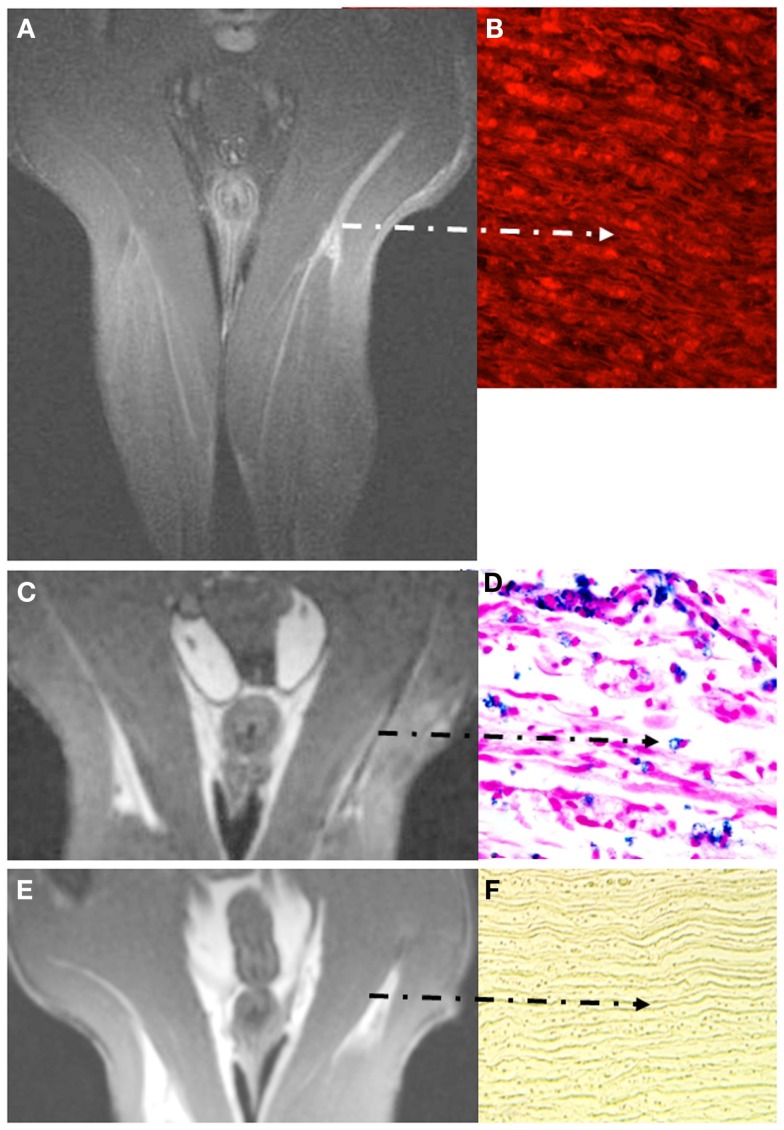
**Visualization of breakdown of the blood nerve barrier (BNB) (A,B) and inflammation in experimental nerve crush (C–F)**. Coronal images depict the pelvis and both thighs of a rat lying in prone position with both legs positioned in a round surface coil (CISS sequence; slice thickness 1 mm). Note that Gf accumulates in the degenerating distal stump on T1-w MRI [arrow in **(A)**] and binds to peripheral nerve structures as revealed by fluorescence of carbocyanine-labeled Gf **(B)**. Gf enhancement ceases not until successful regeneration (not shown). By contrast to breakdown of the BNB, macrophage infiltration is restricted to the early phase of Wallerian degeneration. Five days after sciatic nerve crush focal signal loss is present at the lesion site and distally due to the invasion of SPIO-labeled macrophages from the blood **(C)**. The corresponding paraffin section stained for iron confirms the infiltration of numerous iron-laden macrophages in the degenerating nerve segment **(D)**. At day 8, macrophage infiltration is restricted again to the lesion site and ceases thereafter **(E)**. Correspondingly, distal nerve segments no longer show iron-positive cells after application of SPIO as shown for day 10 in **(F)**. The BNB, however, is still leaky at that time (not shown) indicating that macrophage infiltration occurs within a narrow time window and persistent BNB disturbance does not *per se* induce cellular infiltration. Adapted from Bendszus et al. (2005b); **(A,B)** and Bendszus and Stoll (2003) **(C–F)**.

Nerve degeneration after traumatic injury is accompanied by breakdown of the BNB (Seitz et al., 1989; Bouldin et al., 1991), but, surprisingly, injured nerves do not regularly show Gd-DTPA enhancement (reviewed in Stoll et al., 2009a). Contrastingly, the process of nerve degeneration and subsequent recovery can be assessed by Gf enhanced MRI (Bendszus et al., 2005b) due to the fact that the BNB is disturbed during the degeneration process, but sealed again upon arrival of regenerating nerve sprouts from the proximal stump. Forty-eight hours after crush injury of sciatic nerves in male Wistar rats Gf accumulated within the entire nerve and its lower leg branches undergoing WD and persisted until successful regeneration (Figures 4A,B). Likewise, intense nerve enhancement was present after chronic constriction injury leading to less severe axonal damage. Within several weeks Gf uptake gradually declined from proximal to distal parts of the injured nerve in parallel to regrowth of nerve fibers. Thus, Gf enhanced MRI holds promise to bridge the current diagnostic gap between nerve injury and completed regeneration. Moreover, since non-regenerating, permanently transected nerves exhibit persistent Gf enhancement (Bendszus et al., 2005b), Gf enhanced MRI might help to discern the need for surgical nerve release or grafting if spontaneous regeneration fails. Consequently, Liao et al. (2012) successfully applied Gf enhanced MRI to monitor nerve regeneration after implantation of chitosan nerve conduits with mesenchymal stem cells in a rat model of neurotmesis.

## Cellular MRI

### SPIO/USPIO enhanced MRI

Gadolinium-DTPA and Gf enhanced MRI are crude indicators of BBB or BNB disturbances, but they do not specifically visualize cellular immune responses. Inflammation, however, plays a major role in disorders of the nervous system. While under healthy conditions immune cell access to the nervous system is restricted, leukocytes readily cross the BBB or BNB in the context of pathophysiological processes and enter the neural tissue guided by cell adhesion molecules and chemokines (Man et al., 2007).

There is an ongoing controversy whether immune cell invasion is linked to BBB disruption or occurs independently (see below). A prerequisite for solving this issue are imaging tools that allow monitoring of cell migration *in vivo*. From a clinical perspective, tracking of inflammatory cells could moreover help to identify active phases of CNS and PNS inflammation and to monitor the efficacy of therapeutic interventions. Thus, it is not surprising that cellular neuroimaging has become a field of intense research effort. The preferentially applied contrast agents for cellular MRI are iron oxide nanoparticles and lately, PFC (see Perfluorocarbon Enhanced ^19^F MRI). Depending on their hydrodynamic particle size iron nanoparticles are classified into superparamagnetic iron oxide particles (SPIO; 50–200 nm diameter) and their ultra and very small variants (USPIO; ∼35 nm diameter; VSOP < 10 nm diameter). They all possess relatively large negative magnetic susceptibilities, featuring a more extensive shortening of T1 and T2 relaxation times than Gd-DTPA. Hence, their sensitivity is much higher than that of Gd compounds. The easiest and safest method of iron labeling is spontaneous uptake by blood-borne cells after systemic contrast agent application (Bulte, 2009). If thereafter labeled cells are attracted to a target organ they can be visualized by MRI. Importantly, the property to phagocytose and to migrate to sites of inflammation turns macrophages into ideal targets for cellular MRI. Apart from macrophage phagocytosis SPIO/USPIO are cleared from the circulation by cells of the reticuloendothelial system (RES) in liver and spleen. The extent of cellular labeling in relation to clearance by the RES depends on iron oxide particle size and the net charge of the polymer coating (Hoehn et al., 2007). Safety concerns for both, SPIO and USPIO compounds are little. A recent meta-analysis of 37 phase I to III clinical trials with ferumoxtran-10, an USPIO agent, revealed back pain, pruritus, headache, and urticaria as the most frequent adverse events (Bernd et al., 2009). They are usually mild and of short duration (Anzai et al., 2003). Similarly, SPIO have been associated with low human toxicity. In numerous clinical trials they were shown to cause neither cardiovascular side effects nor clinically relevant laboratory changes (Bluemke et al., 2003; Reimer and Balzer, 2003).

#### EAE and MS

Several proof-of-principle studies have demonstrated that USPIO and SPIO enhanced MRI allow visualization of macrophage infiltration in EAE (Dousset et al., 1999; Rausch et al., 2003; Floris et al., 2004). Brochet et al. (2006) showed that rats with USPIO positive lesions at the first attack suffer from more severe clinical affection and extensive axonal damage at the second EAE bout. This implies that the extent of macrophage infiltration in early EAE may predict the ensuing disease course. Interestingly, in a follow-up study by the same group severely affected rats with USPIO enhancement at the onset of disease showed an imbalanced, proinflammatory macrophage activation profile, both in CNS lesions and the peripheral blood (Mikita et al., 2011). Moreover, macrophage invasion spread from the upper spinal cord and brainstem to the cerebellum and subcortical regions during disease progression. When USPIO were injected in the recovery phase no signal abnormalities were observed indicating that macrophage infiltration in EAE is a timely restricted event that clearly depends on the disease stage. In addition, iron-enhanced MRI was applied as a non-invasive tool for preclinical treatment surveillance. Deloire et al. (2004) demonstrated that macrophage recruitment to the EAE lesions detected by USPIO enhanced MRI is not fully blocked under therapy with natalizumab, a VLA4-antagonist that is in frequent use in patients with relapsing-remitting MS. Moreover, the efficacy of Fingolimod, an oral drug that was recently approved for MS treatment, was successfully monitored by USPIO enhanced MRI in EAE (Rausch et al., 2004).

In MS it is a common conception that the poor clinico-radiological association may be explained in part by diffuse inflammatory activity in the so-called normal appearing white matter (NAWM) which is concealed behind an intact or repaired BBB (Barkhof, 2002; Kutzelnigg et al., 2005). Indeed, microscopic inflammation within the NAWM not amenable by conventional MRI seems to contribute more strongly to disability than the T2 lesion load (Parry et al., 2002; Traboulsee et al., 2003). Thus, it was not surprising that the application of iron-enhanced MRI in EAE and the transfer of this technology to MS patients in small trials have provided insights into CNS inflammation that exceed conventional Gd-DTPA enhanced MRI. Vellinga et al. (2008) detected 188 USPIO positive cerebral lesions in 14 patients with active relapsing-remitting MS. Interestingly, the vast majority of USPIO positive lesions (144/188) showed no concomitant Gd-DTPA enhancement. Furthermore, none of the three different types of USPIO enhancement (i) focal lesions (ii) “return to isointensity” lesions and (iii) ring-enhancing lesions was particularly related to Gd-DTPA uptake (Vellinga et al., 2008). A more recent clinical trial by the same group suggested USPIO as a potential marker for diffuse inflammation in the NAWM of relapsing-remitting and primary progressive MS patients (Vellinga et al., 2009). T1 histogram and region-of-interest analysis in the NAWM showed diffuse T1-shortening after USPIO injection in 16 MS patients indicative of subtle inflammatory activity, but not in gender- and age-matched healthy controls.

#### Ischemic stroke

It has long been established that cerebral ischemia induces a profound inflammatory response involving neutrophils, T-cells, and macrophages (Stoll et al., 1998). In the simple model of photothrombotic infarction (see above), entry of phagocytic hematogenous macrophages is delayed by several days and peaks around days 6–9 after lesion induction (Schroeter et al., 1997). Accordingly, SPIO-induced signal alterations did not occur until day 5 despite persistent breakdown of the BBB in PT lesions. However, at day 6 a hypointense rim appears followed by signal loss in more central areas reflecting the influx of hematogenous macrophages as confirmed by histological analysis (Kleinschnitz et al., 2005). Macrophage inflammation could also be visualized by SPIO/USPIO enhanced MRI after transient or permanent middle cerebral artery occlusion (MCAO), but results are more heterogeneous and conflicting. Some groups found contrast-induced signal loss in the lesion boundary 24 h after permanent MCAO (Rausch et al., 2001). In a transient MCAO model, Denes et al. (2007) did not find USPIO-related signal loss within the first 3 days after stroke induction. Moreover, neither focal signal intensity changes nor iron-positive macrophages were detected in the ischemic hemisphere of Wistar rats when USPIO were applied at a subacute stage 6 days after MCAO (Farr et al., 2011). By contrast, Kim et al. (2008) found areas of signal loss in SPIO enhanced MRI 3 days after reperfusion that corresponded to the accumulation of iron-laden macrophages. A possible explanation for these contradictory results is that the labeling efficacy of monocytes by USPIO in comparison to larger nanoparticles such as SPIO is rather low (Oude Engberink et al., 2007). Moreover, the timing of the contrast agent application and the subsequent MRI appears to have critical impact on the processes depicted by iron-enhanced MRI. Hence, especially in the early infarct phase, focalized signal alterations might rather be caused by trapping of the iron particles in the vasculature than phagocyte infiltration (Desestret et al., 2009).

Several open-label pilot trials with USPIO enhanced MRI were conducted in patients with ischemic stroke. Saleh et al. (2004) applied USPIO in a series of 10 patients 5–6 days after stroke onset. MRI scans were performed 24 and 48 h after USPIO infusion. As principal finding two distinct USPIO-related signal changes were observed: blood pool effects that appeared as signal loss on T2/T2_*_-w images and decreased from the first to the second scan as well as parenchymal contrast enhancement on T1-w images that increased over time and was attributed to macrophage infiltration. When applied early (24–36 h after stroke) in a subsequent study, USPIO enhancement was spatially heterogeneous and only present in a minority of patients (Saleh et al., 2007). Nighoghossian et al. (2007) recruited patients with anterior circulation stroke and administered USPIO 6 days after admission. Three days later, 9 of 10 patients showed USPIO enhancement in the brain parenchyma. Interestingly, whilst most patients featured mild Gd-DTPA uptake, the patient with the most severe BBB breakdown did not exhibit USPIO enhancement.

#### Nerve trauma

Traumatic injury to peripheral nerves similarly induces a profound inflammatory response with macrophage infiltration (Stoll et al., 1989) that leads to rapid removal of myelin debris and a growth-promoting cellular and molecular milieu. Thus, WD is the prototype of a tissue protective M2 type macrophage response (Ydens et al., 2012). SPIO enhanced MRI helped to define the kinetics of macrophage entry into the degenerating nerve segments which starts around day 1 or 2 at the lesion site and extends to the entire distal stump within the first 2 weeks after injury (Bendszus and Stoll, 2003; Figures 4C,D). Accumulation of SPIO-laden macrophages was visible as focal signal loss on T2-w MRI. When SPIO particles were applied 10 days after crush or later degenerating nerves did no longer exhibit signal loss (Figure 4E) despite the presence of numerous myelin-laden macrophages in the endoneurium. There are two possible explanations for this finding: (i) SPIO-based MRI depicts active migration of macrophages from the circulation into nerves, and lack of signal at later stages of WD indicates no further macrophage recruitment (Figure 4F). (ii) Alternatively, myelin-loaded macrophages no longer phagocytose iron particles within the injured nerves. We favor the first possibility since the dynamics of macrophage infiltration shown by SPIO enhanced MRI closely resembles the local expression pattern of macrophage attracting chemokines, which ceases around day 10 (Toews et al., 1998; Tofaris et al., 2002). Moreover, our data suggests that simple leakage of SPIO particles through a defective BNB does not significantly change the intrinsic nerve MR signal, at least in the PNS. These results were recently confirmed in a rodent model of radicular pain. Seven days after transient dorsal root compression T2*–w MRI showed significant iron-induced signal alterations in the nerve roots of the injured, but not of the sham-operated group after systemic application of SPIO (Thorek et al., 2011).

### Perfluorocarbon enhanced ^19^F MRI

Despite its excellent sensitivity iron particle based cellular MRI has several shortcomings. These include false-positive results caused by hemorrhages, blood pool effects and, in some instances, (depending on the half-time of the compound) passive diffusion via a defective BBB or BNB. Additionally, the large ^1^H background signal from mobile water renders unambiguous detection of labeled cells *in vivo* difficult especially if their biodistribution is unclear. Recently, fluorine (^19^F) MRI has emerged as an alternative approach for cellular imaging (Stoll et al., 2012; Temme et al., 2012). ^19^F markers exhibit favorable MR imaging characteristics, such as a magnetic sensitivity close to the proton nucleus and high natural abundance. Moreover, because of the lack of endogenous ^19^F-containing molecules in the body signals originating from injected ^19^F compounds are specific. Another virtue of ^19^F MRI is that it can be performed in a quantitative manner. Ideal fluorine tracers should provide a high payload of ^19^F nuclei. PFC compounds fulfill this requirement and thus were established as tracers for ^19^F MRI in recent years. In animal studies PFC agents were generally well-tolerated (Ebner et al., 2010). Several PFC compounds have also been elaborately studied as artificial blood substitutes (Spahn et al., 2002). In one of these clinical trials cerebral hemorrhages occurred after cardiopulmonary bypass surgery but thorough analysis of the safety data revealed that the study conduct, and not the PFC emulsion itself was responsible for the adverse events (Riess, 2006). Moreover, extensive analysis disclosed no perturbation of hemostasis or blood viscosity after i. v. treatment with PFC that could be related to the observed bleeding tendency (Riess, 2006).

Flogel et al. (2008) were the first to show that systemically injected ^19^F emulsions are efficiently phagocytosed by circulating monocytic cells. Moreover, in mice with myocardial infarction they were able to monitor a time-dependent PFC accumulation in the infarct area. It is well known that myocardial ischemia induces an inflammatory response dominated by cells of the monocyte/macrophage system. Consistently, rhodamine-labeled PFC allowed the identification of ^19^F-positive cells within the infarction as macrophages. Subsequently, *in vivo* ^19^F MRI was successfully applied to monitor immune cell responses in mice with LPS-induced pneumonia (Ebner et al., 2010), abscess formation (Hertlein et al., 2011), and in models of acute allograft rejection (Flogel et al., 2011; Hitchens et al., 2011). However, studies using ^19^F MRI in the nervous system are sparse. Probably due to the low accumulation of PFC compounds in inflammatory lesions of the CNS/PNS compared to other organ systems sensitivity is a major concern (Stoll et al., 2012). Lately, we established ^19^F MRI to depict macrophage infiltration in a rat model of focal inflammatory peripheral nerve injury (Weise et al., 2011). Focal injection of lysolecithin chemically dissolves myelin sheaths and, thereby, elicits a strong inflammatory response within the demyelinated nerve segment (Griffin et al., 1990). *In vivo* MRI 5 days after sciatic nerve damage revealed massive migration of ^19^F labeled cells to the injured nerve section. Intraneural application of saline to the contralateral nerve provoked a slight inflammatory reaction restricted to the perineurium which could also be visualized by ^19^F MRI. However, quantification of the signal strength by *ex vivo* ^19^F spectroscopy indicated a significantly higher number of fluorine-labeled cells in the lysolecithin-damaged nerve than in the contralateral control. Attempts to visualize macrophage responses in EAE by ^19^F MRI *in vivo* failed so far, most likely due to insufficient sensitivity (reviewed in Stoll et al., 2012). Thus, despite its superiority in specificity over SPIO/USPIO-based MRI, better coils and more sensitive MRI sequences are needed for further *in vivo* application of ^19^F MRI in the nervous system.

## The Relation between Breakdown of the BBB/BNB and Cellular Infiltration

There is an ongoing debate whether leakage of the BBB for soluble factors simultaneously provides an unrestricted access of inflammatory cells to the CNS. Histological data from chronic neurodegenerative diseases and EAE already challenged this notion by showing that leukocytes infiltrated the perivascular space without concomitant BBB leakage (Perry et al., 1997; Engelhardt and Wolburg, 2004). Using serial section electron microscopy Wolburg et al. (2005) revealed that mononuclear cells traverse cerebral microvessels by a transcellular pathway, leaving the endothelial tight junctions intact. Moreover, diffusion of hydrophilic molecules and leukocyte recruitment into the CNS take place at distinct sites of the cerebral vascular tree. While the diffusion barrier for solutes is formed by specialized endothelial cells at the level of capillaries leukocyte extravasation usually occurs in the post-capillary segments (Bechmann et al., 2007; Engelhardt and Sorokin, 2009).

The advent of novel MR imaging techniques contributed to the partial solution of this controversy. In the first paragraphs we described contrast agents allowing assessment of BBB/BNB dysfunction (Gd-DTPA, Gf M) as well as SPIO/USPIO enhanced MRI to monitor macrophage infiltration. Combined use of these MR contrast agents in individual animals and selected patients disclosed that breakdown of the BBB/BNB and macrophage infiltration can occur independently: in other words inflammatory cells can cross the BBB without acutely disturbing the BBB, and, vice versa, long-lasting disruption of the BBB/BNB does not necessarily entertain permanent cellular infiltration.

### EAE and MS

First MR evidence that migration of inflammatory cells to the nervous system is not compulsorily associated with BBB/BNB opening arose from experiments in EAE animals. Rausch et al. (2003) described EAE lesions exhibiting either USPIO or Gd-DTPA enhancement. This mismatch was most prominent during the first relapse when large numbers of USPIO enhancing lesions did not show any Gd-DTPA uptake. Floris et al. (2004) claimed that impairment of the BBB (as shown by Gd-DTPA enhancement) preceded monocyte infiltration (as assessed by USPIO enhancement) in EAE thereby implicating a firm sequence of events in lesion formation. This view was later on challenged by others (Bendszus et al., 2005a; Berger et al., 2006). These studies described USPIO/SPIO positive lesions in areas with an intact BBB suggestive for a different pattern of lesion evolution. Interestingly, in the latter study USPIO enhancing lesions disappeared after the acute inflammatory attack, while areas with BBB damage recovered more slowly (Berger et al., 2006).

As discussed above Gd-DTPA enhancement underestimates the number of lesions with breakdown of the BBB. We therefore took advantage of the more sensitive MR contrast agent Gf and compared the number and location of Gf and SPIO enhancing lesions in individual rats with EAE (Ladewig et al., 2009). Numerous Gf positive lesions appeared in the spinal cord, brain stem, and optic nerves and roughly a similar number of lesions showed signal loss after SPIO application indicative for macrophage infiltration. However, the spatial distribution of the lesions was completely different with almost no overlap (Figures 2E,F). These findings provide further evidence that macrophages can enter the CNS leaving the BBB intact (SPIO positive, Gf negative lesions) and that breakdown of the BBB (Gf positive lesions) is not necessarily associated with cellular inflammation (SPIO positive lesions). At present it is unclear how these processes interact, e.g., whether cellular infiltration evokes BBB leakage with a delay or vice versa transient disturbances of the BBB predetermine sites of later cellular invasion in EAE.

Though Gf has not yet been developed for human application, clinical studies in MS patients using Gd-DTPA strongly support the results obtained in EAE animals. Dousset et al. (2006) performed a Gd-DTPA/USPIO enhanced MRI study in a cohort of 10 relapsing-remitting MS patients and found that 31 out of 57 lesions were collectively enhanced with both contrast agents. Importantly, 24 Gd-DTPA enhancing lesions were USPIO negative while two USPIO lesions did not show Gd-DTPA uptake. Vellinga et al. (2008) compared USPIO enhanced MRI to the longitudinal pattern of Gd-DTPA enhancement in 19 relapsing-remitting MS patients. They found that 77% of USPIO positive lesions were located in areas with an intact BBB. Moreover, USPIO enhancing lesions were more abundant than Gd-DTPA enhancing lesions and remained visible for longer time periods than Gd-DTPA. In 4% of USPIO positive lesions USPIO enhancement preceded Gd-DTPA uptake by several weeks. Just recently, Tourdias et al. (2012) longitudinally assessed disease activity with combined Gd-DTPA and USPIO enhanced MRI in 10 relapsing patients and 14 patients with progressive MS over a 6 month period. In this study, the use of both contrast agents considerably increased the diagnostic yield enabling the detection of 51% more lesions than with Gd-DTPA alone. USPIO enhancement was also observed in patients with a progressive disease course lacking Gd-DTPA enhancement (Tourdias et al., 2012). Thus, these studies unanimously support the assumption that SPIO/USPIO and Gd-DTPA enhanced MRI cover different aspects of MS pathophysiology and activity.

### Ischemic stroke

Several MRI studies conducted in experimental stroke also indicate that BBB opening and cellular inflammation are not necessarily linked. In the PT model it was shown that SPIO-laden macrophages enter the lesion at a subacute stage (around day 6) while BBB breakdown occurs immediately after lesion evolution and persists for weeks (Kleinschnitz et al., 2003, 2005). Beyond that, macrophages were still abundant in the infarction at later time points but did not show any iron uptake (Kleinschnitz et al., 2003). In MCAO models available data is more controversial. While some studies failed to show iron-induced signal loss in the infarction despite prolonged opening of the BBB and infiltration of neutrophils (Denes et al., 2007), others suggest that USPIO penetrate in the CNS as free particles over a disrupted barrier (Desestret et al., 2009). Thus, there is an ongoing controversy to what extent USPIO enhancement corresponds to leukocyte infiltration or passive leakage through a defective BBB.

Most importantly, diverse signatures of conventional Gd-DTPA and iron-enhanced MRI persisted when the application of USPIO was transferred to patients with ischemic stroke. Saleh et al. (2004) found USPIO-related signal changes in the subacute infarct stage of ten stroke patients, while Gd-DTPA enhancement occurred in only six of them. In another study, Nighoghossian et al. (2007) confirmed spatial discrepancies between USPIO-related signal alterations and BBB breakdown assessed by Gd-DTPA in patients with anterior circulation stroke.

### Nerve trauma

The conception that breakdown of barriers in the nervous system is not necessarily congruent with cellular infiltration is further reinforced by studies in nerve trauma. Axotomy or crush of a peripheral nerve leads to degeneration of the distal nerve segment (WD) accompanied by a rapid breakdown of the BNB as shown by extravasation of albumin in histological studies (Seitz et al., 1989; Bouldin et al., 1991). Accordingly, degenerating nerves show continuous Gf enhancement throughout WD which terminates not until successful regeneration is accomplished (Bendszus et al., 2005b). Upon SPIO enhanced MRI a different picture emerges (Bendszus and Stoll, 2003). SPIO-induced signal loss indicative of macrophage infiltration starts at the lesion site within 2 days, extends distally within the first week after injury and suddenly ceases after 10 days (Figure 4). Thus, SPIO application later than 10 days after nerve injury is not further accompanied by signal alterations on T2-w MRI despite the fact that the BNB is still defective. Functionally, these findings indicate that, despite persistent breakdown of the BNB for up to 4 weeks after injury (when nerve regeneration is completed), there is no continuous macrophage invasion at the late phase of WD and regeneration. The pattern of macrophage invasion revealed by SPIO enhanced MRI very well corresponded to the local expression of chemokines in the degenerating nerve supporting the notion that the local molecular environment, but not the simple breakdown of the BNB is responsible for the attraction of inflammatory cells.

## Conclusion

The ability to visualize nervous tissue by MRI has revolutionized clinical neurology during the last three decades, but the signal alterations seen in diseases are mainly non-specific. The use of Gd-DTPA as a MR contrast agent allows detection of tumors and areas with a disturbed BBB, however, sensitivity is limited. In an attempt to depict molecular and cellular processes more precisely novel contrast agents have been developed. In experimental studies, Gf allows more sensitive assessment of disturbances of the BBB and BNB than Gd-DTPA. Thereby, the diagnostic yield is highly increased in animal models of MS, functional and transient changes of BBB properties hitherto undisclosed can be assessed and, finally, the process of nerve regeneration which is linked to sealing of the BNB, can be accurately followed. Cellular contrast agents such as iron-containing SPIO/USPIO or PFC allow tracking of inflammatory responses by MRI, mainly macrophage infiltration into the NS. By combining both imaging technologies it became increasingly clear that breakdown of the BBB/BNB and leukocyte infiltration are distinct processes showing much less overlap than previously anticipated. Further clinical development of these MR contrast agents is warranted since they hold promise to provide unique insights into the pathophysiology and dynamics of inflammatory disorders of the NS and could improve treatment surveillance.

## Conflict of Interest Statement

The authors declare that the research was conducted in the absence of any commercial or financial relationships that could be construed as a potential conflict of interest.
